# The effect of the **H**^−1^ scaling factors *τ* and *ω* on the structure of **H** in the single-step procedure

**DOI:** 10.1186/s12711-018-0386-x

**Published:** 2018-04-13

**Authors:** Johannes W. R. Martini, Matias F. Schrauf, Carolina A. Garcia-Baccino, Eduardo C. G. Pimentel, Sebastian Munilla, Andres Rogberg-Muñoz, Rodolfo J. C. Cantet, Christian Reimer, Ning Gao, Valentin Wimmer, Henner Simianer

**Affiliations:** 1 KWS SAAT SE, Einbeck, Germany; 2 Departamento de Producción Animal, Facultad de Agronomía, Universidad de Buenos Aires, Buenos Aires, Argentina; 3 Institute of Animal Breeding, Bavarian State Research Center for Agriculture, Poing‑Grub, Germany; 4 CONICET, Buenos Aires, Argentina; 5 Facultad de Ciencias Veterinarias, IGEVET - Instituto de Genética Veterinaria (UNLP-CONICET LA PLATA), La Plata, Argentina; 6 INPA, UBA-CONICET, Buenos Aires, Argentina; 7 Animal Breeding and Genetics Group, Center for Integrated Breeding Research, University of Goettingen, Goettingen, Germany; 8 National Engineering Research Center for Breeding Swine Industry, Guangdong Provincial Key Lab of Agro-animal Genomics and Molecular Breeding, College of Animal Science, South China Agricultural University, Guangzhou, China

## Abstract

**Background:**

The single-step covariance matrix **H** combines the pedigree-based relationship matrix ${\mathbf {A}}$ with the more accurate information on realized relatedness of genotyped individuals represented by the genomic relationship matrix ${\mathbf {G}}$. In particular, to improve convergence behavior of iterative approaches and to reduce inflation, two weights $\tau$ and $\omega$ have been introduced in the definition of ${\mathbf {H}}^{-1}$, which blend the inverse of a part of ${\mathbf {A}}$ with the inverse of ${\mathbf {G}}$. Since the definition of this blending is based on the equation describing ${\mathbf {H}}^{-1}$, its impact on the structure of ${\mathbf {H}}$ is not obvious. In a joint discussion, we considered the question of the shape of ${\mathbf {H}}$ for non-trivial $\tau$ and $\omega$.

**Results:**

Here, we present the general matrix ${\mathbf {H}}$ as a function of these parameters and discuss its structure and properties. Moreover, we screen for optimal values of $\tau$ and $\omega$ with respect to predictive ability, inflation and iterations up to convergence on a well investigated, publicly available wheat data set.

**Conclusion:**

Our results may help the reader to develop a better understanding for the effects of changes of $\tau$ and $\omega$ on the covariance model. In particular, we give theoretical arguments that as a general tendency, inflation will be reduced by increasing $\tau$ or by decreasing $\omega$.

## Background

A genomic relationship matrix ${\mathbf{G}}$ provides information on the realized relatedness of individuals but requires genotyping, which increases the costs of breeding programs. Thus, breeders are often confronted with the situation that not all individuals for which expected relatedness can be derived from the pedigree are genotyped. The single-step approach [1–3] is a practical way to combine these two different sources of information—the pedigree relationship matrix ${\mathbf {A}}$ and the genomic relationship matrix ${\mathbf {G}}$—in one matrix ${\mathbf {H}}$. This relationship matrix ${\mathbf {H}}$ relates all individuals as does ${\mathbf {A}}$, but incorporates the more accurate information provided by ${\mathbf {G}}$. Here, the central concept is to substitute entries of ${\mathbf {A}}$ by the corresponding entries of ${\mathbf {G}}$ and to adapt the remaining relationships accordingly. In more detail, the matrix ${\mathbf {H}}$ is defined by

1 $$\begin{aligned} {\mathbf {H}} := {\mathbf {A}} + \begin{pmatrix} {\mathbf {A}}_{12}{\mathbf {A}}_{22}^{-1} ({\mathbf {G}}-{\mathbf {A}}_{22}) {\mathbf {A}}_{22}^{-1} {\mathbf {A}}_{21} &{} \quad {\mathbf {A}}_{12} {\mathbf {A}}_{22}^{-1} ({\mathbf {G}}-{\mathbf {A}}_{22}) \\ ({\mathbf {G}}-{\mathbf {A}}_{22}){\mathbf {A}}_{22}^{-1}{\mathbf {A}}_{21} &{} \quad ({\mathbf {G}}-{\mathbf {A}}_{22}) \\ \end{pmatrix}. \end{aligned}$$

Here, the individuals are divided into two groups: Group 1 contains the individuals whose genotype is not available and Group 2 consists of the genotyped individuals. Thus, ${\mathbf {A}}_{11}$ denotes the entries of ${\mathbf {A}}$ that provide the relationships within Group 1, ${\mathbf {A}}_{12}$ and ${\mathbf {A}}_{21}$ the relationships between the individuals of the two groups, and ${\mathbf {A}}_{22}$ the pedigree relationships within Group 2. Moreover, ${\mathbf {A}}_{22}^{-1}$ denotes the inverse of ${\mathbf {A}}_{22}$, which is not in general identical to the bottom-left block of ${\mathbf {A}}^{-1}$, i.e. $({\mathbf {A}}^{-1})_{22}$. With this definition, we have substituted the inner group pedigree relationship of Group 2 by the genomic relationship, which means ${\mathbf {H}}_{22}={\mathbf {G}}$. The terms ${\mathbf {A}}_{12} {\mathbf {A}}_{22}^{-1} ({\mathbf {G}}-{\mathbf {A}}_{22})$ adapt the relationships within Group 1 and the relationships between individuals of the two groups according to the changed relationships within Group 2 to generate a positive semi-definite, valid covariance structure (this transfer of information can be also interpreted in terms of imputation [4, 5]).

Since many applications use the inverse of a relationship matrix, Eq. (1) is usually written on the level of its inverse (see [3] and equations 18, 19 of [6]):

2 $$\begin{aligned} {\mathbf {H}}^{-1} = {\mathbf {A}}^{-1}+ \begin{pmatrix}0 &{} \quad 0 \\ 0 &{} \quad ({\mathbf {G}}^{-1}-{\mathbf {A}}_{22}^{-1}) \\ \end{pmatrix} \end{aligned}.$$

Based on this setup, several previous papers have discussed the question of how to combine ${\mathbf {A}}$ and ${\mathbf {G}}$ optimally. In this context, approaches which have been followed adapt ${\mathbf {G}}$ to ${\mathbf {A}}$ [7, 8] or conversely ${\mathbf {A}}$ to ${\mathbf {G}}$ [8–10]. Moreover, two scaling factors $\tau$ and $\omega$ have been introduced [11, 12]:

3 $$\begin{aligned} {\mathbf {H}}_{\tau ,\omega }^{-1} : = \mathbf {A}^{-1}+ \begin{pmatrix}0 &{} \quad 0 \\ 0 &{} \quad (\tau {\mathbf {G}}^{-1}-\omega {\mathbf {A}}_{22}^{-1}) \\ \end{pmatrix} \end{aligned}.$$

The main purposes of the introduction of these parameters were to ensure convergence of iterative approaches that address the mixed models [11], and to reduce inflation of predictions [13]. Compared to methods based on ${\mathbf {A}}$ or ${\mathbf {G}}$ alone, these issues have been assumed to be enhanced by inconsistencies between ${\mathbf {A}}$ and ${\mathbf {G}}$ [14], and this blending is one possibility among several to approach the problem [15].

Equation (3) is defined on the level of ${\mathbf {H}_{\tau ,\omega }^{-1}}$, but the effect of the introduction of $\omega$ and $\tau$ on the shape of ${\mathbf {H}}$ is not obvious. In particular, breeders aiming at implementing the single-step method in breeding programs raised the question of how these parameters affect the relationship model ${\mathbf {H}}_{\tau ,\omega }$. Here, we present ${\mathbf {H}}_{\tau ,\omega }$ in a general form, as a matrix dependent on $\tau$ and $\omega$ and discuss some of its properties. Moreover, we provide arguments for a reduction in inflation of predicted breeding values being expected when $\tau$ increases or when $\omega$ decreases. Finally, to set a contrast to the widely used cattle data [12, 13, 16, 17], we screened for optimal values of $\tau$ and $\omega$ with respect to predictive ability, inflation and iterations to convergence on a well investigated, publicly available wheat data set [18]. Our results may help to develop an understanding for the effects on the covariance model when these parameters are changed. In particular, this may be of interest for people who aim at implementing the single-step method with non-trivial parameters $\tau$ and $\omega$ in practical breeding programs.

## ${\mathbf {H}}_{\tau ,\omega }$ and some particular choices of $\tau$ and $\omega$

We will first describe ${\mathbf {H}}_{\tau ,\omega }$ and discuss some special cases. Mathematical arguments for the presented statements are provided in the “Appendix”. If an inverse of a matrix is used, the implicit assumption on invertibility is made (also if not mentioned explicitly). In particular, ${\mathbf {A}}$ is considered invertible on account of its construction from the pedigree (granted clones are absent) [19].

### **Central statement**

The inverse of ${\mathbf {H}}_{\tau ,\omega }^{-1}$ defined by Eq. (3) is

4 $$\begin{aligned} {\mathbf {H}}_{\tau ,\omega } = {\mathbf {A}} + \begin{pmatrix} {\mathbf {A}}_{12}{\mathbf {A}}_{22}^{-1} \; ({\mathbf {H}}_{22} - {\mathbf {A}}_{22}) \; {\mathbf {A}}_{22}^{-1} {\mathbf {A}}_{21}&{} \quad {\mathbf {A}}_{12} {\mathbf {A}}_{22}^{-1} \; ({\mathbf {H}}_{22} - {\mathbf {A}}_{22}) \\ ({\mathbf {H}}_{22} - {\mathbf {A}}_{22}) \; {\mathbf {A}}_{22}^{-1}{\mathbf {A}}_{21} &{} \quad ({\mathbf {H}}_{22} - {\mathbf {A}}_{22}) \\ \end{pmatrix} \end{aligned}$$

with

5 $$\begin{aligned} {\mathbf {H}}_{22}=\left( \tau {\mathbf {G}}^{-1} + (1-\omega ){\mathbf {A}}^{-1}_{22}\right) ^{-1}. \end{aligned}$$

The structure of Eq. (4) is identical to that of Eq. (1), but with ${\mathbf {G}}$ substituted by Eq. (5). Considering ${\mathbf {H}}_{22}$, we see that the parameterization of the weights $\omega$ and $\tau$ is “reverse” in the sense that $\tau$ and $\omega$ appear with opposite signs in front of them. In particular, this implies that ${\mathbf {H}}_{\tau ,\omega }$ is not necessarily positive semi-definite when $\omega > 1$ since this leads to a negative factor for ${\mathbf {A}}_{22}^{-1}$ and thus has to be compensated by $\tau {\mathbf {G}}^{-1}$ to give a positive semi-definite matrix. However, positive semi-definiteness of ${\mathbf {H}}_{\tau ,\omega }$ is guaranteed, if ${\mathbf {G}}$ and ${\mathbf {A}}$ are positive definite and $\tau \ge 0$ and $\omega \le 1$, but not both at their boundary, that is not $\tau =0$ and $\omega =1$ at the same time.

### **Lemma 1**

*Let* ${\mathbf {A}}$ *and* ${\mathbf {G}}$ *be positive definite and let* $\tau \ge 0$ *and* $\omega \le 1$, *but not* $\tau =0=1-\omega$. *Then* ${\mathbf {H}}_{\tau ,\omega }$ *is positive semi-definite*.

Note that due to the “reverse parameterization” in form of weights $(1-\omega )$ and $\tau$ in Eq. (5), the sets of parameter values, which guarantee positive semi-definiteness of the single-step matrix ${\mathbf {H}}_{\tau ,\omega }$, are distinct. If both $\tau$ and $(1-\omega )$ are positive, then positive semi-definiteness of ${\mathbf {H}}_{\tau ,\omega }$ is guaranteed. In particular, this also means that a negative $\omega$ gives a valid covariance model. Thus, a grid to test combinations would be rather within $(\tau , \omega ) \in [0,2] \times [-1,1]$ than $(\tau , \omega ) \in [0,2] \times [0,1]$, which has often been the frame for the choice of parameters [13, 16, 17].

In the following, we will discuss special choices of $\tau$ and $\omega$.

(i) If $\tau = \omega = 1$, we are dealing with the original single-step method of Eq. (2).
(ii) If $\tau = \omega = 0$, then ${\mathbf {H}}_{22}= {\mathbf {A}}_{22}$ and thus ${\mathbf {H}}={\mathbf {A}}$.
(iii) If $\omega = \tau = \lambda > 0$, then ${\mathbf {H}}_{22}=\left( \lambda {\mathbf {G}}^{-1} + (1-\lambda ){\mathbf {A}}_{22}^{-1}\right) ^{-1}.$
(iv) If $\omega =1$, then ${\mathbf {H}}_{22}= \tau ^{-1} {\mathbf {G}}$.
(v) If $$\tau =1$$, then $${\mathbf {H}}_{22}= \left( {\mathbf {G}}^{-1} + (1-\omega ){\mathbf {A}}_{22}^{-1}\right) ^{-1}.$$

Case (i) is already obvious on the level of ${\mathbf {H}}^{-1}$, but it can also be seen on the level of ${\mathbf {H}}_{\tau ,\omega }$ that Eq. (4) coincides in this case with Eq. (1), since ${\mathbf {H}}_{22}= {\mathbf {G}}$. If instead $\tau =\omega =0$ as for case (ii) then ${\mathbf {H}}_{0,0} = {\mathbf {A}}$ and the single-step BLUP becomes the traditional pedigree-BLUP. Also note that case (iii), for which $\tau$ and $\omega$ are equal, has already been addressed in [3] and results in a weighted harmonic mean of ${\mathbf {G}}$ and ${\mathbf {A}}_{22}$.

In case (iv) in which $\omega$ is equal to 1, ${\mathbf {H}}_{22}=\tau ^{-1}{\mathbf {G}}$. With increasing $\tau$, the entries of ${\mathbf {H}}_{12}$, ${\mathbf {H}}_{21}$, ${\mathbf {H}}_{22}$ will shrink towards ${\mathbf {0}}$ and ${\mathbf {H}}_{11}$ to the *Schur complement* ${\mathbf {A}}/{\mathbf {A}}_{22}:={\mathbf {A}}_{11}-{\mathbf {A}}_{12} {\mathbf {A}}_{22}^{-1} {\mathbf {A}}_{21}$.

In case (v), we see that if we fix $\tau =1$, ${\mathbf {H}}_{22}$ is not significantly simplified. Moreover, since the weighted sum of ${\mathbf {A}}^{-1}$ and ${\mathbf {G}}^{-1}$ is inverted in ${\mathbf {H}}_{22}$, the factor $(1-\omega )$ may also introduce a weight on the entries of ${\mathbf {G}}$. We see that this is indeed the case with the following example. Choosing $(\tau ,\omega )=(1,0.5)$,

$$\begin{aligned} \text{ with } \qquad {\mathbf {G}}= \begin{pmatrix} 2 &{} \quad 1 \\ 1 &{} \quad 2 \end{pmatrix}, \qquad \text{ and } \qquad {\mathbf {A}}_{22}= \begin{pmatrix} 1 &{} \quad 0 \\ 0 &{} \quad 1 \end{pmatrix}, \qquad \text{ gives } \qquad {\mathbf {H}}_{22}= \begin{pmatrix} 0.9\overline{33}&{} \quad 0.2\overline{66} \\ 0.2\overline{66} &{} \quad 0.9\overline{33} \end{pmatrix}. \end{aligned}$$

In this example, the non-diagonal elements of ${\mathbf {A}}_{22}$ are 0, but the non-diagonal elements of ${\mathbf {H}}_{22}$ deviate from the corresponding entries of ${\mathbf {G}}$ which are equal to 1. Thus, $\omega$ cannot be interpreted as being only a weight of the pedigree contribution ${\mathbf {A}}_{22}$ to the covariance ${\mathbf {H}}_{22}$.

## The effect of $\tau$ and $\omega$ on inflation

A main purpose of the introduction of these parameters is the reduction of inflation of the predicted breeding values [13, 16] which is manifested and diagnosed by a slope $b < 1$ in a regression of observed values (y-axis) on predictions (x-axis). Please recall here that the regression of observed values on predictions should be preferred to a regression of predictions on observed values for model evaluation [20]. We will argue why—as a general tendency—increasing $\tau$ or decreasing $\omega$ may lead to a reduced inflation.

In many models used for animal and plant breeding, the genetic component ${\mathbf {g}}$ is modeled as a random variable with multivariate normal distribution, zero mean and structured variance, for instance given by $\sigma _{\mathbf {g}}^2 {\mathbf {H}}_{\tau ,\omega }$ in single-step. The simplest version without a fixed effect can be written as:

6 $$\begin{aligned} {\mathbf {y}}= {\mathbf {g}}+ {\mathbf{\epsilon }}\end{aligned},$$

where ${\mathbf {y}}$ denotes the $n \times 1$ vector of phenotypes, ${\mathbf {g}}\sim {\mathscr {N}}(0,\sigma _{\mathbf {g}}^2 {\mathbf {H}}_{\tau ,\omega })$ the genetic effect and ${\mathbf{\epsilon }}\sim {\mathscr {N}}(0,\sigma _{\mathbf{\epsilon }}^2{\mathbf {I}}_n)$ the independent and identically distributed errors. The best linear unbiased prediction (BLUP [19, 21]) for this ${\mathbf {g}}$ is given by

7 $$\begin{aligned} {\hat{\mathbf {g}}}=\left( {\mathbf {I}}+ \frac{\sigma _{\mathbf{\epsilon }}^2}{\sigma _{\mathbf {g}}^2}{\mathbf {H}}_{\tau ,\omega }^{-1}\right) ^{-1} {\mathbf {y}}\end{aligned}.$$

We will apply some results on positive semi-definite matrices to this model and its BLUP to show when a change in the values of $\tau$ and $\omega$ (to $\tau '$ and $\omega '$) reduces the variance of the estimate of the genetic component. In the following, we use the partial order on the positive semi-definite matrices (the so-called Löwner order [22]), to speak about variance “increase” and “reduction” in a multivariate context. For two positive semi-definite matrices ${\mathbf {K}}_1$ and ${\mathbf {K}}_2$, ${\mathbf {K}}_1 \succeq {\mathbf {K}}_2$ if and only if ${\mathbf {K}}_1 - {\mathbf {K}}_2$ is positive semi-definite. With this notation, ${\mathbf {K}}_1 \succeq {\mathbf {0}}$ means that ${\mathbf {K}}_1$ is positive semi-definite. For a reference on the properties of the Löwner order see [23].

### **Lemma 2**

*Let* ${\mathbf {A}}$ *and* ${\mathbf {G}}$ *be positive definite and* ${\mathbf {H}}_{\tau ,\omega }$ *as introduced*.

- *Let* $\tau \le \tau '$ *and* $\omega \ge \omega '$ *be given such that* $\left( {\mathbf {H}}_{\tau ,\omega } \right) _{22 } \succeq 0 \preceq \left( {\mathbf {H}}_{\tau ',\omega '} \right) _{22 }$ . *Then*
  $$\begin{aligned} \left( {\mathbf {H}}_{\tau ,\omega } \right) _{22 } \succeq {\left( {\mathbf {H}}_{\tau ',\omega } \right) _{22 }} \succeq \left( {\mathbf {H}}_{\tau ',\omega '} \right) _{22 } \text{ and } \left( {\mathbf {H}}_{\tau ,\omega } \right) _{22 } \succeq {\left( {\mathbf {H}}_{\tau ,\omega '} \right) _{22 }} \succeq \left( {\mathbf {H}}_{\tau ',\omega '} \right) _{22 } \end{aligned}.$$
- *Moreover*,
  $$\begin{aligned} \left( {\mathbf {H}}_{\tau ,\omega } \right) _{22 } \succeq \left( {\mathbf {H}}_{\tau ',\omega '} \right) _{22 } \Longleftrightarrow {\mathbf {H}}_{\tau ,\omega } \succeq {\mathbf {H}}_{\tau ',\omega '} \end{aligned}.$$
- *For two matrices of the shape of the BLUP solution of Eq*. (7)
  $$\begin{aligned} {\mathbf {K}}_1:=\left( {\mathbf {I}}+ \lambda {\mathbf {H}}_{\tau ,\omega }^{-1} \right) ^{-1} \; \text{ and } \; {\mathbf {K}}_2:=\left( {\mathbf {I}}+ \lambda {\mathbf {H}}_{\tau ',\omega '}^{-1} \right) ^{-1} \end{aligned},$$
  *with a *$\lambda > 0$, *we have*
  $$\begin{aligned} {\mathbf {H}}_{\tau ,\omega } \succeq {\mathbf {H}}_{\tau ',\omega '} \Longleftrightarrow {\mathbf {K}}_1 \succeq {\mathbf {K}}_2 \end{aligned}.$$

Lemma 2(a) illustrates that if we keep $\tau$ constant and decrease $\omega$ to $\omega '$, the resulting matrix $\left( {\mathbf {H}}_{\tau ,\omega '} \right) _{22}$ will be “smaller” with respect to the Löwner order. The same is true if we keep $\omega$ constant and increase $\tau$ to $\tau '$. Part (b) transfers this observation to the level of ${\mathbf {H}}_{\tau ,\omega }$. Finally, part (c) connects ${\mathbf {H}}_{\tau ,\omega }$ with the BLUP of model (6).

We now illustrate how this reduction with respect to the Löwner order, transfers to the variance of breeding value estimates ${\hat{\mathbf {g}}}$ in this simple model of ${\hat{\mathbf {g}}}:=\left( {\mathbf {I}}+ \lambda {\mathbf {H}}_{\tau ,\omega }^{-1} \right) ^{-1} {\mathbf {y}}$.

### **Proposition 1**

*Let* ${\mathbf {K}}_1\succeq {\mathbf {K}}_2$, ${\mathbf {K}}_1 {\mathbf {K}}_1\succeq {\mathbf {K}}_2 {\mathbf {K}}_2$, *and let* ${\hat{\mathbf {g}}}_i := {\mathbf {K}}_i {\mathbf {y}}$ *be the corresponding estimate of the breeding values. Moreover, let the empirical mean of both estimates be the same* ${\mathbf {E}}({\hat{\mathbf {g}}}_1)={\mathbf {E}}({\hat{\mathbf {g}}}_2)$ *and let* ${\text {Var}}({\hat{\mathbf {g}}}_i)$ *denote the empirical variance of the vector* ${\hat{\mathbf {g}}}_i$, *defined by*

$$\begin{aligned} \frac{1}{n} \sum \limits _{j=1}^{n} {\hat{g}}_{i,j}^2 - {\mathbf {E}}({\hat{\mathbf {g}}}_i)^2. \end{aligned}$$

Then

$$\begin{aligned} {\text {Var}}({\hat{\mathbf {g}}}_1) \ge {\text {Var}}({\hat{\mathbf {g}}}_2) \end{aligned}.$$

Proposition 1 illustrates that an important effect of using an $\omega$ smaller than 1, or a $\tau$ larger than 1 may be the reduction of the variance of the predicted genetic values. To see this, recall that Lemma 2(a) and (b) stated that reducing $\omega$ to $\omega '$ and keeping $\tau$ fixed implies ${\mathbf {H}}_{\tau ,\omega } \succeq {\mathbf {H}}_{\tau ,\omega '}$. The same is true for increasing $\tau$ to $\tau '$ with fixed $\omega$. Lemma 2(c) then implies that ${\mathbf {K}}_1 \succeq {\mathbf {K}}_2$. Thus, provided that all preconditions are given, Proposition 1 states that the variance of the estimated breeding values is reduced.

The critical assumption is ${\mathbf {K}}_1{\mathbf {K}}_1\succeq {\mathbf {K}}_2{\mathbf {K}}_2$, since this is not implied by ${\mathbf {K}}_1 \succeq {\mathbf {K}}_2$ (for a counter example see [24]). Thus, this will not be totally satisfied in practice. Instead, because we are dealing with a partial order, often neither ${\mathbf {K}}_1{\mathbf {K}}_1\succeq {\mathbf {K}}_2{\mathbf {K}}_2$ nor ${\mathbf {K}}_2{\mathbf {K}}_2\succeq {\mathbf {K}}_1{\mathbf {K}}_1$ will hold, but the difference of the two products may result in an indefinite matrix (i.e. one with both positive and negative eigenvalues). However, if only a few eigenvalues of the difference ${\mathbf {K}}_1{\mathbf {K}}_1 - {\mathbf {K}}_2{\mathbf {K}}_2$ are smaller than zero, this assumption will be correct to a good approximation. Moreover, also the assumption of $\mathbf {E}({\hat{\mathbf {g}}}_1)=\mathbf {E}({\hat{\mathbf {g}}}_2)$ will only approximately hold in practice. Finally, recall that the variance components are usually estimated and an adapted estimate can compensate the effects of changes of the parameters $\tau$ and $\omega$.

We will give an example of how a reduced empirical variance may reduce inflation.

### *Example 1*

Let $\mathbf {y}$ be a vector of measured data and $\mathbf {g}_1:=\mathbf {K}_1 \mathbf {y}$ with $\mathbf {K}_1\succeq \mathbf {0}$. Moreover, let $\mathbf {g}_2:=0.5 \mathbf {K}_1 \mathbf {y}$ which means $\mathbf {K}_2=0.5\mathbf {K}_1$. Then $\mathbf {K}_1 \succeq \mathbf {K}_2$ and $\mathbf {K}_1\mathbf {K}_1 \succeq \mathbf {K}_2\mathbf {K}_2$ and ${\text {Var}}(\mathbf {g}_2) = 0.25{\text {Var}}(\mathbf {g}_1)$.

Defining the inflation as *b* of an ordinary least squares regression of $\mathbf {y}$ on $\mathbf {g}$

$$\begin{aligned} \mathbf {y}= \alpha + b \mathbf {g}+ {\mathbf{\epsilon }}\end{aligned}$$

gives $b_2=\frac{{\text {Cov}}(\mathbf {y},\mathbf {g}_2)}{{\text {Var}}(\mathbf {g}_2)}=\frac{0.5}{0.25} b_1= 2 b_1$. Note here that a value of $b> 1$ means that the estimates of the breeding values are deflated and $b < 1$ that they are inflated. Thus $b_2> b_1>0$ means that the inflation is reduced when $\mathbf {K}_2$ is used instead of $\mathbf {K}_1$.

Example 1 illustrates that the reduced variance of the predicted genetic values may reduce inflation. It is worth highlighting that the scaling factor used in this example was formulated on the level of $\mathbf {K}_i$ which does not simply translate to a scaled variance component for $\mathbf {H}_{\tau ,\omega }$. In the next section, we give a small example with a well investigated wheat data set [18].

## An example with wheat data

We assessed predictive ability, inflation and number of iterations up to convergence with varying parameters $\tau$ and $\omega$ on a publicly available wheat data set [18, 25]. The aim was to seek for the optimal combinations of both parameters, which maximize the predictive ability or minimize the inflation or the number of iterations to convergence, respectively. Moreover, we were interested in the general behavior of inflation when $\tau$ and $\omega$ are varied.

### Data

The data set which we used consists of 599 CIMMYT wheat lines, genotyped with 1279 Diversity Array Technology markers indicating whether a certain allele is present (1) or not (0) in the respective line. The lines were grown in four different environments and grain yield was recorded for each line and each environment (for more details see [18]). We used only the phenotypic data of environment 1 for our comparisons. To see whether the choice of which lines are considered as (not) genotyped has a significant impact on properties of the single-step procedure, we split the lines into two parts according to the order in the data set and considered two scenarios: In scenario 1 (hereinafter referred to as SC1), lines 1 to 300 were treated as not genotyped and the remaining lines 301 to 599 were used as genotyped group. Thus, the pedigree relationship of lines 301 to 599 represents $\mathbf {A}_{22}$ and their genomic relationship represents $\mathbf {G}$. The genomic relationship matrix was calculated according to VanRaden [26]: $\mathbf {G}= (\mathbf {Z}-\mathbf {P})(\mathbf {Z}-\mathbf {P})^T/ \sum _{j=1}^{p} p_j (1-p_j)$, with $\mathbf {Z}$ denoting the $n \times p$ matrix giving the states of the *p* markers of the *n* individuals, and $\mathbf {P}$ denoting the matrix with identical rows giving the column averages of $\mathbf {Z}$. The same procedure was repeated in scenario 2 (hereinafter referred to as SC2) but the genotyped group consisted of lines 1 to 300. Note again that the order was used as provided by the data set.

### Parameter grid

To seek for the optimal values for both parameters, 420 combinations of $\tau$ and $\omega$ were tested for each scenario. This number of combinations resulted from varying both parameters on a grid defined by 0.10 steps dividing the interval $[-1,1]$ for $\omega$, or [0.1, 2] for $\tau$. To evaluate the performance of each parameter combination, we constructed $\mathbf {H}_{\tau ,\omega }^{-1}$ by Eq. (3) for each combination of the parameters. Consequently, 420 different $\mathbf {H}_{\tau ,\omega }^{-1}$ matrices were calculated in R [27] and transferred to the blupf90 software [28] to estimate the breeding values using the single-step procedure.

### Evaluation of the prediction

To evaluate the predictions obtained with the different matrices, a cross-validation was run by partitioning the 599 wheat lines into 10 disjoint groups of approximately 60 lines each (regardless of whether their genomic information had been used in the single-step covariance matrix). The partitions used were those provided with the data set, which had been generated randomly [18]. Iteratively, each group was used as a test set and models were fit with the remaining lines. Prediction quality was evaluated for these 60 lines in terms of predictive ability and inflation. The former was measured as Pearson’s correlation between the phenotype and the estimated breeding value (EBV) for the test set. Inflation was calculated as the coefficient of regression of the phenotype on the EBV (for the test set). The optimal combination of parameter values should have a regression coefficient close to 1 (neither inflation nor deflation). The number of iterations to convergence was also recorded.

### Results

**Fig. 1:**
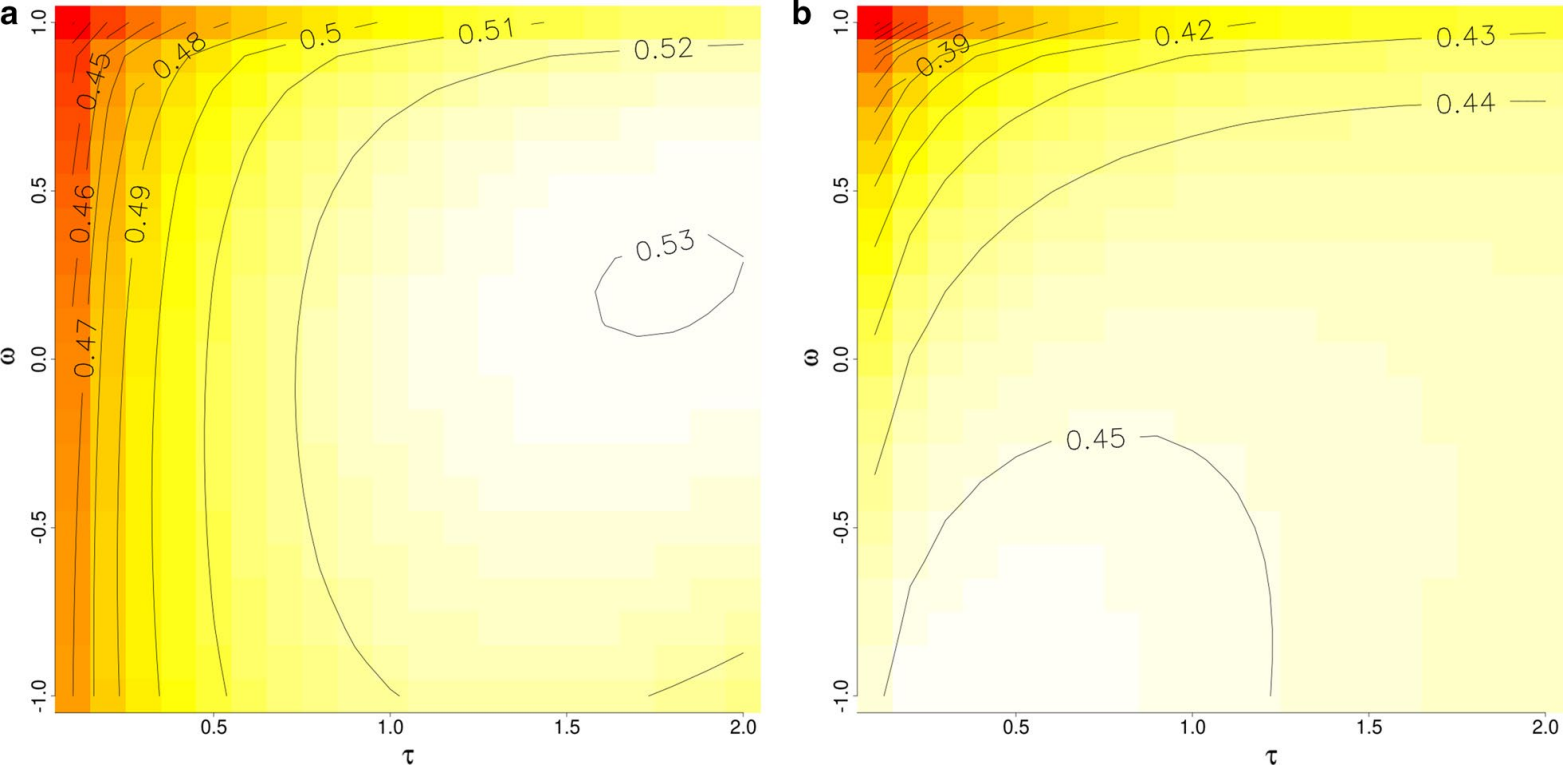
Heat plots for predictive ability calculated as the Pearson’s correlation between phenotype and EBV for each combination of parameters $\tau$ and $\omega$ for **a** SC1 and **b** SC2. The lighter the colour, the higher the predictive ability of the corresponding $(\tau ,\omega )$ combination

Figure 1 illustrates the average predictive ability obtained for different choices of $(\tau ,\omega )$ for the two different scenarios SC1 and SC2. The pedigree BLUP predictive ability is given by $(\tau ,\omega )=(0,0)$. The closest here is $(\tau ,\omega )=(0.1,0)$ with a predictive ability of 0.46 for the first scenario and 0.43 for the second one and which is in accordance with the value of 0.448 originally reported [18]. The maximum predictive ability for SC1 was obtained with $(\tau ,\omega )=(1.8,0.2)$ whereas in SC2 it was reached with $(\tau ,\omega )=(0.4,-1.0)$. The location of the maximum differs, but in both scenarios we observe a broad optimum, that is a plateau on which the predictive ability hardly changes. An important observation is that the maximal predictive ability is very different between the two scenarios (0.53 vs. 0.45).

**Fig. 2:**
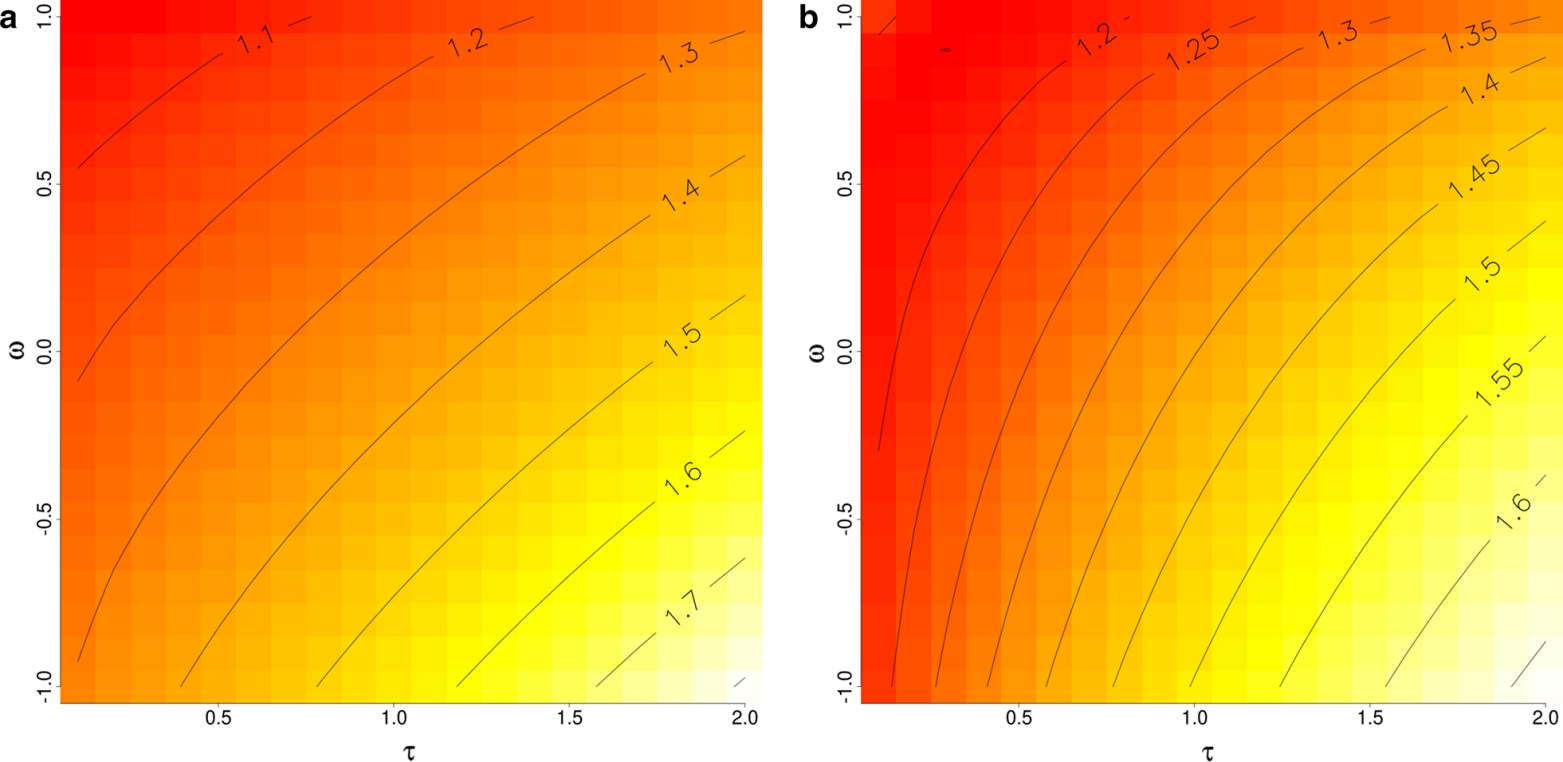
Heat plots for inflation calculated as the slope in the regression of observed phenotypes on predictions for **a** SC1 and **b** SC2. The lighter the colour, the higher the slope and lower the inflation

Figure 2 shows the mean inflation for each considered $(\tau ,\omega )$ combination for the two scenarios. The combinations with the lowest inflation, that is the highest regression coefficient *b* were $(\tau ,\omega )=(2,-1)$ in both scenarios, as suggested by our theoretical results. We see the tendency that both, increasing $\tau$ or decreasing $\omega$ reduces inflation in the sense of increasing *b*. However, note that in our example, we are already in a situation of deflation and reducing the variance of ${\hat{\mathbf {g}}}$ increases the predictive bias.

Lastly, the optimal values of the parameters in terms of a minimal number of iterations to convergence were $(\tau ,\omega )=(1.9,0.8)$ for SC1 and $(\tau ,\omega )=(1.0,-\,0.8)$ for SC2. However, for most combinations, the number of iterations was between 26 and 32 which indicates that the influence of $(\tau ,\omega )$ on the number of iterations required is limited for this data set (results not shown).

## Discussion

Here we presented the general form of the single-step relationship matrix $\mathbf {H}_{\tau ,\omega }$, when blending parameters $\tau$ and $\omega$ are defined on the level of its inverse [11, 12]. The matrix obtained (Eq. 4) is similar to the original single-step relationship matrix (Eq. 1) but with the role of $\mathbf {G}$ replaced by expression Eq. (5). Moreover, we discussed some special choices of these parameters including the case for which $\tau$ and $\omega$ are equal, which was also the first adjustment of $\mathbf {H}$ discussed in the literature [3].

The reduction in inflation was one of the main motivations for using the blending parameters [13, 16]. We illustrated with theoretical considerations that increasing $\tau$ or decreasing $\omega$ tends to reduce the empirical variance of ${\hat{\mathbf {g}}}_i$, which again may lead to a reduced inflation. Our theoretical arguments are limited by their assumptions, but should hold to a good approximation. To reinforce these results with an empirical exploration, we gave a small example with a well investigated wheat data set [18]. There, the pattern observed for inflation was largely in accordance with what we expected from our theoretical considerations. With regard to predictive ability, the parameters showed broad optimality and varied strongly across the two scenarios SC1 and SC2. Both observations may be data set specific and the latter a consequence of the small population size.

Finally, note that similar effects on inflation can also be achieved with other methods as for instance by explicitly reducing the additive variance or by accounting for inbreeding [5] (see in this context also Example 1). It may be worth considering the single-step method in more detail from a theoretical perspective to address the causes of inflation. Recent studies reported results in this direction by for instance attributing inflation to inconsistencies between genomic and pedigree relationships and by suggesting that accounting for inbreeding and unknown parent groups in a proper way may reduce this problem [5]. Moreover, it has also been highlighted that selective genotyping and selective imputation may have an impact on the properties of ssBLUP [29].

## Conclusion

We provided theoretical arguments that increasing $\tau$ or decreasing $\omega$ may mainly decrease inflation by decreasing the variance of the estimated breeding values ${\hat{\mathbf {g}}}$. Alternative solutions that address the problems of single-step predictions from a more theoretical point of view may be found by investigating the consistency problems of $\mathbf {A}$ and $\mathbf {G}$ with respect to scaling and coding further.

## Appendix

### Derivation of $\mathbf {H}_{\tau ,\omega }$

Given that we know that Eq. (1) is the inverse of Eq. (2), we define

$$\begin{aligned} \mathbf {H}_{22}^{-1}:= \left( \tau \mathbf {G}^{-1} + (1- \omega )\mathbf {A}_{22}^{-1} \right) \end{aligned}$$

Then, we can rewrite Eq. (3) as

8 $$\begin{aligned} \mathbf {H}_{\tau ,\omega }^{-1} : = \mathbf {A}^{-1}+ \begin{pmatrix}0 &{} \quad 0 \\ 0 &{} \quad (\tau \mathbf {G}^{-1}-\omega \mathbf {A}_{22}^{-1})\end{pmatrix} = \mathbf {A}^{-1}+ \begin{pmatrix}0 &{} \quad 0 \\ 0 &{} \quad (\mathbf {H}_{22}^{-1}- \mathbf {A}_{22}^{-1})\\ \end{pmatrix} \end{aligned}$$

The right-hand side has the structure of Eq. (2), and thus plugging $\mathbf {H}_{22}$ at the corresponding positions of $\mathbf {G}$ in Eq. (1) gives Eq. (4). $\square$

### Lemma 1: positive semi-definiteness of $\mathbf {H}_{\tau ,\omega }$

For a block-partitioned matrix:

$$\begin{aligned} \mathbf {M}= \begin{bmatrix} \mathbf {M}_{11}&\quad \mathbf {M}_{12} \\ \mathbf {M}_{21}&\quad \mathbf {M}_{22} \end{bmatrix} \end{aligned}$$

We recall that if $\mathbf {M}_{22}$ is non-singular, then the matrix $\mathbf {M}_{11} - \mathbf {M}_{12}\mathbf {M}_{22}^{-1}\mathbf {M}_{21}$ is called the *Schur complement* of $\mathbf {M}_{22}$ in $\mathbf {M}$ and denoted as $\mathbf {M}/\mathbf {M}_{22}$. The Schur complement has interpretations as a conditional covariance matrix, and possesses useful properties [30]. In particular, Theorem 1.12(b) of the book by Zhang [31] states that for $\mathbf {M}_{22}$ non-singular

9 $$\begin{aligned} \mathbf {M}\succeq \mathbf {0} \Longleftrightarrow \left( \mathbf {M}_{22} \succ \mathbf {0} \text{ and } \mathbf {M}/\mathbf {M}_{22} \succeq \mathbf {0} \right) \end{aligned}$$

Here, $\succ$ and $\succeq$ denote positive (semi-)definiteness. Now, consider $\mathbf {H}/\mathbf {H}_{22}$,

$$\begin{aligned} \mathbf {H}/\mathbf {H}_{22}&= \mathbf {H}_{11} - \mathbf {H}_{12}\mathbf {H}_{22}^{-1}\mathbf {H}_{21} \\&= \mathbf {A}_{11} + \mathbf {A}_{12}\mathbf {A}_{22}^{-1}(\mathbf {H}_{22} - \mathbf {A}_{22})\mathbf {A}_{22}^{-1}\mathbf {A}_{21} - \mathbf {H}_{12}\mathbf {H}_{22}^{-1}\mathbf {H}_{21} \\&= \mathbf {A}_{11} + \mathbf {A}_{12}\mathbf {A}_{22}^{-1}(\mathbf {H}_{22} - \mathbf {A}_{22})\mathbf {A}_{22}^{-1}\mathbf {A}_{21} - \mathbf {A}_{12}\mathbf {A}_{22}^{-1}\mathbf {H}_{22}\mathbf {A}_{22}^{-1}\mathbf {A}_{21} \\&= \mathbf {A}_{11} + \mathbf {A}_{12}\mathbf {A}_{22}^{-1}(\mathbf {H}_{22} - \mathbf {A}_{22} - \mathbf {H}_{22})\mathbf {A}_{22}^{-1}\mathbf {A}_{21} \\&= \mathbf {A}_{11} - \mathbf {A}_{12}\mathbf {A}_{22}^{-1}\mathbf {A}_{21} \\&= \mathbf {A}/\mathbf {A}_{22} \end{aligned}$$

As $\mathbf {A}\succeq 0$, Eq. (9) states that $\mathbf {A}/\mathbf {A}_{22} = \mathbf {H}/\mathbf {H}_{22} \succeq \mathbf {0}$, and thus $\mathbf {H}\succeq \mathbf {0} \Longleftrightarrow \mathbf {H}_{22} \succ \mathbf {0}$, which in turn is the case when $\tau \ge 0$ and $\omega \le 1$, but not $\tau =0=1-\omega$, due to the presupposed positive definiteness of $\mathbf {G}$ and $\mathbf {A}_{22}$. $\square$

### Lemma 2: variance reduction with varying $\tau$ and $\omega$

To prove (a), we consider the case of $\tau \le \tau '$ and $\omega =\omega '$. The case of $\tau = \tau '$ and $\omega \ge \omega '$ is analogous. We will need at several steps the properties of positive semi-definite matrices and the partial order “$\preceq$” (*c.f.* [23]).

$$\begin{aligned} &\left( \mathbf {H}_{\tau ,\omega }\right) _{2,2} \succeq \left( \mathbf {H}_{\tau ',\omega }\right) _{2,2}\Longleftrightarrow \left( \tau \mathbf {G}^{-1} + (1-\omega ) \mathbf {A}_{22}^{-1} \right) ^{-1} \succeq \left( \tau ' \mathbf {G}^{-1}+ (1-\omega )\mathbf {A}_{22}^{-1} \right) ^{-1} \\&\quad \Longleftrightarrow \left( \tau \mathbf {G}^{-1} + (1-\omega ) \mathbf {A}_{22}^{-1} \right) \preceq \left( \tau ' \mathbf {G}^{-1}+ (1-\omega )\mathbf {A}_{22}^{-1} \right) \\&\quad \Longleftrightarrow \tau \mathbf {G}^{-1} \preceq \tau ' \mathbf {G}^{-1} \\&\quad \Longleftrightarrow \tau '-\tau \ge 0 \\&\quad \Longleftrightarrow \tau ' \ge \tau \end{aligned}$$

To prove (b), we first write $\mathbf {H}_{\tau ,\omega }$ as $\mathbf {A}+\mathbf {W}^T((\mathbf {H}_{22})_{\tau ,\omega }-\mathbf {A}_{22})\mathbf {W}$ with

$$\begin{aligned} \mathbf {W}= \begin{bmatrix} \mathbf {A}_{22}^{-1} \mathbf {A}_{21}&\quad \mathbf {I}\end{bmatrix} \end{aligned}$$

Then

$$\begin{aligned} (\mathbf {H}_{22})_{\tau ,\omega } \succeq (\mathbf {H}_{22})_{\tau ',\omega '}&{\mathop {\Longleftrightarrow }\limits ^{*}} \mathbf {W}^T(\mathbf {H}_{22})_{\tau ,\omega }\mathbf {W}\succeq \mathbf {W}^T(\mathbf {H}_{22})_{\tau ',\omega '}\mathbf {W}\\&\Longleftrightarrow \mathbf {A}+\mathbf {W}^T(\mathbf {H}_{22})_{\tau ,\omega }\mathbf {W}\succeq \mathbf {A}+\mathbf {W}^T(\mathbf {H}_{22})_{\tau ',\omega '}\mathbf {W}\\&\Longleftrightarrow \mathbf {H}_{\tau ,\omega } \succeq \mathbf {H}_{\tau ',\omega '} \end{aligned}$$

To see $*$, recall that positive semi-definiteness of a matrix $\mathbf {A}$ is defined by $\forall \mathbf {y}: \mathbf {y}^T \mathbf {A}\mathbf {y}\ge 0$. Define ${\tilde{\mathbf {y}}}:=\mathbf {W}\mathbf {y}$ to prove “$\Rightarrow$”. Conversely, to prove “$\Leftarrow$”, we need to show that $\mathbf {W}^T(\mathbf {H}_{22})_{\tau ,\omega }\mathbf {W}\succeq \mathbf {W}^T(\mathbf {H}_{22})_{\tau ',\omega '}\mathbf {W}$ implies $(\mathbf {H}_{22})_{\tau ,\omega }\succeq (\mathbf {H}_{22})_{\tau ',\omega '}$. This is true, since the rank of $\mathbf {W}$ is the number of genotyped individuals $n_2$ (due to $\mathbf {I}$ being included in the definition of $\mathbf {W}$). This means that for any chosen $\mathbf {y}\in \mathbb {R}^{n_2}$, we find an inverse image ${\tilde{\mathbf {y}}} \in \mathbb {R}^{n_1 +n_2}$ such that $\mathbf {y}= \mathbf {W}{\tilde{\mathbf {y}}}$. Thus, for any $\mathbf {y}$, use the representation ${\tilde{\mathbf {y}}}^T\mathbf {W}^T(\mathbf {H}_{22})_{\tau ,\omega }\mathbf {W}{\tilde{\mathbf {y}}} \ge {\tilde{\mathbf {y}}}^T \mathbf {W}^T(\mathbf {H}_{22})_{\tau ',\omega '}\mathbf {W}{\tilde{\mathbf {y}}}$ to get $\mathbf {y}^T(\mathbf {H}_{22})_{\tau ,\omega } \mathbf {y}\ge \mathbf {y}^T (\mathbf {H}_{22})_{\tau ',\omega '}\mathbf {y}$.

To prove (c), we consider:

$$\begin{aligned} \mathbf {H}_{\tau ,\omega } \succeq \mathbf {H}_{\tau ',\omega '}&\Longleftrightarrow \mathbf {H}_{\tau ,\omega }^{-1} \preceq \mathbf {H}_{\tau ',\omega '}^{-1} \\&\Longleftrightarrow \mathbf {I}+\lambda \mathbf {H}_{\tau ,\omega }^{-1} \preceq \mathbf {I}+\lambda \mathbf {H}_{\tau ',\omega '}^{-1} \\&\Longleftrightarrow (\mathbf {I}+\lambda \mathbf {H}_{\tau ,\omega }^{-1})^{-1} \succeq (\mathbf {I}+\lambda \mathbf {H}_{\tau ',\omega '}^{-1})^{-1} \\&\Longleftrightarrow \mathbf {K}_1 \succeq \mathbf {K}_2 \end{aligned}$$

$$\square$$

### Proposition 1: reducing the empirical variance of the predicted genetic values

Let us define the empirical variance of vector ${\hat{\mathbf {g}}}_i=({\hat{g}}_{i,j})_{j=1,\ldots ,n}$ by

$$\begin{aligned} {\text {Var}}({\hat{\mathbf {g}}}_i):= \frac{1}{n} \sum \limits _{j=1}^{n}\left( {\hat{g}}_{i,j}- \frac{1}{n}\sum \limits _{j=1}^n {\hat{g}}_{i,j} \right) ^2= \frac{1}{n} \sum \limits _{j=1}^{n} {\hat{g}}_{i,j}^2 - \mathbf {E} ({\hat{\mathbf {g}}}_i)^2 \end{aligned}$$

To show that

$$\begin{aligned} {\text {Var}}({\hat{\mathbf {g}}}_1) \ge {\text {Var}}({\hat{\mathbf {g}}}_2) \end{aligned}$$

we have to show that

$$\begin{aligned} \frac{1}{n} \sum \limits _{j=1}^{n} {\hat{g}}_{1,j}^2 - \mathbf {E} ({\hat{\mathbf {g}}}_1)^2 \ge \frac{1}{n} \sum \limits _{j=1}^{n} {\hat{g}}_{2,j}^2 - \mathbf {E} ({\hat{\mathbf {g}}}_2)^2, \end{aligned}$$

which reduces to

$$\begin{aligned} \sum \limits _{j=1}^{n} {\hat{g}}_{1,j}^2 \ge \sum \limits _{j=1}^{n} {\hat{g}}_{2,j}^2 \end{aligned}$$

since the empirical means are assumed to be identical. Using the definition of ${\hat{\mathbf {g}}}_i$ gives

$$\begin{aligned} \sum \limits _{j=1}^{n} {\hat{g}}_{1,j}^2 = {\hat{\mathbf {g}}}_1^T {\hat{\mathbf {g}}}_1 = (\mathbf {K}_1 \mathbf {y})^T (\mathbf {K}_1 \mathbf {y}) = \mathbf {y}^T \mathbf {K}_1 \mathbf {K}_1 \mathbf {y}\ge \mathbf {y}^T \mathbf {K}_2 \mathbf {K}_2 \mathbf {y}= \sum \limits _{j=1}^{n} {\hat{g}}_{2,j}^2 , \end{aligned}$$

which is true due to the initially made assumption of $\mathbf {K}_1 \mathbf {K}_1\succeq \mathbf {K}_2 \mathbf {K}_2$. $\square$
